# Efficacy and Safety of Artemether in the Treatment of Chronic Fascioliasis in Egypt: Exploratory Phase-2 Trials

**DOI:** 10.1371/journal.pntd.0001285

**Published:** 2011-09-06

**Authors:** Jennifer Keiser, Hanan Sayed, Maged El-Ghanam, Hoda Sabry, Saad Anani, Aly El-Wakeel, Christoph Hatz, Jürg Utzinger, Sayed Seif el-Din, Walaa El-Maadawy, Sanaa Botros

**Affiliations:** 1 Department of Medical Parasitology and Infection Biology, Swiss Tropical and Public Health Institute, Basel, Switzerland; 2 University of Basel, Basel, Switzerland; 3 Public Health Department, Theodor Bilharz Research Institute, Giza, Egypt; 4 Hepatogastroenterology Department, Theodor Bilharz Research Institute, Giza, Egypt; 5 Parasitology Department, Theodor Bilharz Research Institute, Giza, Egypt; 6 Ministry of Health and Population, Alexandria Governorate, Alexandria, Egypt; 7 Ministry of Health and Population, Behera Governorate, Behera, Egypt; 8 Department of Medical Services and Diagnostic, Swiss Tropical and Public Health Institute, Basel, Switzerland; 9 Institute for Social and Preventive Medicine, University of Zurich, Zurich, Switzerland; 10 Department of Epidemiology and Public Health, Swiss Tropical and Public Health Institute, Basel, Switzerland; 11 Pharmacology Department, Theodor Bilharz Research Institute, Giza, Egypt; The George Washington University Medical Center, United States of America

## Abstract

**Background:**

Fascioliasis is an emerging zoonotic disease of considerable veterinary and public health importance. Triclabendazole is the only available drug for treatment. Laboratory studies have documented promising fasciocidal properties of the artemisinins (e.g., artemether).

**Methodology:**

We carried out two exploratory phase-2 trials to assess the efficacy and safety of oral artemether administered at (i) 6×80 mg over 3 consecutive days, and (ii) 3×200 mg within 24 h in 36 *Fasciola*-infected individuals in Egypt. Efficacy was determined by cure rate (CR) and egg reduction rate (ERR) based on multiple Kato-Katz thick smears before and after drug administration. Patients who remained *Fasciola*-positive following artemether dosing were treated with single 10 mg/kg oral triclabendazole. In case of treatment failure, triclabendazole was re-administered at 20 mg/kg in two divided doses.

**Principal Findings:**

CRs achieved with 6×80 mg and 3×200 mg artemether were 35% and 6%, respectively. The corresponding ERRs were 63% and nil, respectively. Artemether was well tolerated. A high efficacy was observed with triclabendazole administered at 10 mg/kg (16 patients; CR: 67%, ERR: 94%) and 20 mg/kg (4 patients; CR: 75%, ERR: 96%).

**Conclusions/Significance:**

Artemether, administered at malaria treatment regimens, shows no or only little effect against fascioliasis, and hence does not represent an alternative to triclabendazole. The role of artemether and other artemisinin derivatives as partner drug in combination chemotherapy remains to be elucidated.

## Author Summary

*Fasciola hepatica* and *F. gigantica* are two liver flukes that parasitize herbivorous large size mammals (e.g., sheep and cattle), as well as humans. A single drug is available to treat infections with *Fasciola* flukes, namely, triclabendazole. Recently, laboratory studies and clinical trials in sheep and humans suffering from acute fascioliasis have shown that artesunate and artemether (drugs that are widely used against malaria) also show activity against fascioliasis. Hence, we were motivated to assess the efficacy and safety of oral artemether in patients with chronic *Fasciola* infections. The study was carried out in Egypt and artemether administered according to two different malaria treatment regimens. Cure rates observed with 6×80 mg and 3×200 mg artemether were 35% and 6%, respectively. In addition, high efficacy was observed when triclabendazole, the current drug of choice against human fascioliasis, was administered to patients remaining *Fasciola* positive following artemether treatment. Concluding, monotherapy with artemether does not represent an alternative to triclabendazole against fascioliasis, but its role in combination chemotherapy regimen remains to be investigated.

## Introduction

Fascioliasis, a zoonotic disease caused by a liver fluke infection of the species *Fasciola hepatica* and *F. gigantica,* is of considerable veterinary and public health importance [1], [2]. Owing to global changes, infections with *Fasciola* spp. appear to be emerging or re-emerging in several parts of the world [1]. An estimated 91 million people are at risk of fascioliasis, whereas the estimated number of infections shows a large range from 2.4 to 17 million [3]. Severe clinical complications in the chronic phase of a *Fasciola* infection include cholangitis, cholecystitis, jaundice, and biliary colic [1], [4].

In Egypt, fascioliasis is an important clinical problem, particularly among school-aged children living in rural areas of the Nile Delta [5], [6]. Prevalence rates of *Fasciola* infections have been reduced in recent years, explained by control measures put forth by the Egyptian governorates, including triclabendazole administration [6]. Indeed, chemotherapy with triclabendazole, a member of the benzimidazole family of anthelmintics, is the current mainstay for morbidity control of fascioliasis [7]. It should be noted, however that triclabendazole is often difficult to obtain, since it is currently registered in only four countries for human treatment [7]. In addition, resistant fluke populations have been reported from several countries [7]–[9]. Unfortunately, no vaccine is currently available for prevention of fascioliasis [10].

There is a need to develop new fasciocidal drugs. Several studies have documented that the artemisinins (e.g., artemether and artesunate), which have become the most important antimalarial drugs, particularly when deployed as artemisinin-based combination therapy (ACT) [11], also possess schistosomicidal [12] and fasciocidal activities [13]. Regarding fascioliasis, complete elimination of worms was achieved in rats experimentally infected with adult *F. hepatica* when artesunate and artemether were administered at single oral doses (400 and 200 mg/kg, respectively) 8 weeks postinfection [14]. Severe tegumental changes and death of flukes occurred when *Fasciola* spp. were incubated with an artemisinin derivative (50–100 µg/ml) *in vitro* [14]–[17]. Artesunate and artemether, given by the intramuscular route, yielded high egg and worm burden reductions in natural *F. hepatica* infections in sheep [18], [19]. Finally, a study in 100 Vietnamese patients has shown that artesunate might also play a role in the treatment of acute fascioliasis, as patients treated with artesunate were significantly more likely to be free of abdominal pain when compared to triclabendazole-treated patients [20].

The aim of the present study was to assess the efficacy and safety of oral artemether, adhering to two different malaria treatment regimens [21], [22], in patients with a chronic *Fasciola* spp. infection. The study was carried out in a *Fasciola*-endemic area of Egypt, where *Schistosoma mansoni* co-exists, but malaria is absent.

## Methods

### Ethics Statement

Ethical clearance was obtained from the Theodor Bilharz Research Institute (Giza, Egypt), the Ministry of Health and Population (Cairo, Egypt), and the Ethics Committee of Basel, Switzerland (EKBB, reference no. 54/07). The trial is registered with Current Controlled Trials (reference no. ISRCTN10372301). Written informed consent was obtained from eligible study participants or parents/legal guardians from individuals aged below 16 years.

### Study Design, Sample Size, and Outcome Measures

The study was designed as an interventional, open-label, non-randomized, proof-of-concept trial, consisting of two separate single-arm studies, to evaluate the efficacy and safety of two artemether regimens in the treatment of asymptomatic *Fasciola*-infected patients. Twenty individuals were assigned to each study, following recommendations for pilot studies of at least 12 patients per treatment [23] and sufficient number of patients who might not comply to follow-up.

The primary end points were cure rate (CR, defined as percentage of patients who became *Fasciola* egg-negative after treatment, who were egg-positive at study enrollment) and egg reduction rate (ERR, defined as reduction of geometric mean (GM) egg output after treatment divided by the GM of the same individuals before treatment, multiplied by a factor 100) of *Fasciola* infection, 28 days after the final dosing. Incidence of adverse events, monitored up to 2 days after the final dosing, was used as secondary outcome measure.

Paticipants who remained *Fasciola* positive following artemether treatment were orally treated with a single 10 mg/kg dose of triclabendazole. Efficacy of triclabendazole was determined in the frame of the second intervention study. Patients who were still found with *Fasciola* eggs in their stool following 10 mg/kg triclabendazole were treated with 20 mg/kg triclabendazole in two divided doses.

### Study Area and Population

Study 1 was carried out between April and July 2007 in El-Haddad El-Bahary village, Behera governorate, north-east of Delta. El-Haddad El-Bahary village is s a typical rural setting, with canals fed from the Nile River and no access to the Mediterranean. The total population in the village is 8144.

Study 2 was conducted between August 2008 and May 2010 in Abis village, located south-west of Alexandria. It comprises 10 sub-villages, with an estimated total population of 35,000. Abis village is fed by water canals drawn from the Nile River, with no access to the Mediterranean.

### Treatment

Artemether, formulated as 40 mg capsules (study 1) and 50 mg tablets (study 2) was purchased from Kunming Pharmaceutical Cooperation (Artemidine®; Kunming, People's Republic of China). The following two treatment schemes were investigated: (i) 6×80 mg over 3 consecutive days (study 1) and (ii) 3×200 mg within 24 h (study 2). Treatment was supervised by a physician with date and precise time of drug administration recorded. Patients were observed for 1 h to ensure retention of medication. In case of vomiting or any treatment-related adverse events, a second dose of artemether was administered.

Triclabendazole (Egaten® 250 mg tablets, scored tablets) was the product of Novartis (Basel, Switzerland). Patients who failed to become *Fasciola* egg-negative following artemether administration received 10 mg/kg triclabendazole. The triclabendazole dosage, according to the patients' weight, was calculated in half-tablet increments with a maximum of 2.5 tablets (625 mg). In case of triclabendazole treatment failures (assessed in study 2), patients were provided two doses of 10 mg/kg of triclabendazole given on subsequent days according to manufacturer's instructions.

### Study Flow

Several weeks before conducting a parasitological baseline survey, the health directorate of Beheira (study 1) and Alexandria governorate (study 2) were informed about the objectives, procedures, and potential risks and benefits. After written informed consent was obtained, participants were asked to provide a stool sample in order to screen for the presence of *F. hepatica* and/or *F. gigantica* eggs. Stool collection containers were labeled with patient's name and a unique identifier (ID). Filled containters were transfered to a laboratory for diagnostic work-up. Two additional stool samples were collected on consecutive days among participants who were found with *Fasciola* eggs in their feces. In addition, a blood sample was collected before drug administration to examine hematologic parameters, liver, and kidney functions.

At enrollment a full clinical examination was carried out to assess participants' general health status. Exclusion criteria were: (i) age below 5 years, (ii) pregnancy, (iii) major systemic illnesses (e.g., history of chronic illness such as cancer, diabetes, hypertension, chronic heart, liver or renal disease, severe liver disease of other etiology), and (iv) recent history of anthelmintic treatment (e.g., albendazole, bithionol, dehydroemetine, mebendazole, praziquantel, and triclabendazole taken within the past 4 weeks). Patients meeting our inclusion criteria were treated with artemether, which was administered over 3 consecutive days (study 1) or within 24 h (study 2).

Adverse events were monitored on each treatment day and for 24–48 h following the final dosing. Participants were asked to report any potential drug-related signs and symptoms using a standardized questionnaire. Full clinical examinations were performed on all participants. Adverse events were graded (i.e., mild, moderate, severe, and serious) and recorded. Therapy was offered to patients presenting with adverse events, as judged by the study physician.

Five and 28 days posttreatment, blood samples were collected for clinical chemistry analyses. The final parasitological assessment started on day 28 posttreatment: stool samples were obtained from all study participants over 5 consecutive days. Patients found with *Fasciola* eggs in their stool following artemether administration were treated with 10 mg/kg triclabendazole. In study 2, stool samples were collected from triclabendazole-treated patients 28 days posttreatment over 3 consecutive days and CRs and ERRs were determined. Those patients who remained *Fasciola* positive were retreated with a double dose of triclabendazole (20 mg/kg given 24 h apart) [24] and efficacy (CRs and ERRs) was assessed 28 days posttreatment, on the basis of three stool samples. In both groups of triclabendazole-treated patients, liver and renal function and hematological parameters were determined pre- and posttreatment (5 and 28 days after drug administration).

### Laboratory Procedures

For detection and quantification of *Fasciola* eggs, all stool samples were processed shortly after collection using the Kato-Katz technique [25]. From each stool sample, 3–6 thick smears were prepared on microscope slides. The slides were transported in enumerated boxes to the Theodor Bilharz Research Institute and examined within a maximum of 48 h. The presence of *S. mansoni* and soil-transmitted helminths (i.e., *Ascaris lumbricoides* and *Trichuris trichiura*) was also determined and recorded for each participant individually. Each slide was examined independently in a blind manner by two microscopists. For quality control, several slides were re-examined by a senior staff. For confirmation of *Fasciola* and other helminth eggs, at baseline the merthiolate-iodine formaldehyde (MIF) concentration technique [26] was employed for one stool sample per participant. Briefly, 2.35 ml of stock MIF solution was added to at least about 0.5 g of each stool sample in a 15 ml centrifuge tube, closed with a rubber stopper, and placed in a refrigerator for subsequent examination. On the next morning 0.15 ml of Lugol's iodine solution was added to each tube. After centrifugation, the upper layers of sedimented feces containing parasite material were examined under a microscope.

Laboratory investigations of blood included total leukocyte count, hemoglobin, eosinophilic count, alanine transpeptidase (ALT), aspartate transpeptidase (AST), alkaline phosphatase (ALP), gamma glutamyl transpeptidase (GGT), total serum bilirubin, blood urea, and serum creatinine. The blood specimens were collected into gel serum tubes (for clinical chemistry variables) and EDTA tubes (for hematology variables). Blood specimens collected into gel tubes were centrifuged at 1800–2000 *g* for 10–15 min. All blood specimens were analyzed on the day of collection.

### Statistical Analysis

Data were entered using EpiData version 6.04 (Epidata Association; Odense, Denmark). CR was calculated as proportion of individuals excreting *Fasciola* eggs before treatment and absence of eggs at study end. To determine infection intensity, the number of *Fasciola* eggs per Kato-Katz thick smear (41.7 mg of stool) was multiplied by a factor 24 to obtain eggs per gram of stool (EPG). Fecal egg counts (FECs) of multiple slides per individual were averaged, using the arithmetric mean. To calculate the reduction in infection intensity, individual egg counts were logarithmically transformed (log (count + 1) and the GM expressed as the antilogarithm of the mean. The ERR was calculated as [1 - GM FEC after treatment divided by GM FEC at admission multiplied by a factor 100]. Although infection intensity thresholds are currently lacking for infections with *Fasciola* [27], we classified infections into two groups: (i) light (1–99 EPG) and (ii) moderate/heavy (≥100 EPG). Of note, a threshold of 100 EPG is also used to distringuish between light and moderate (100–399 EPG) and heavy (≥400 EPG) *S. mansoni* infection [27]. Fisher's exact test, including 95% confidence intervals (CI), and Mann-Whitney U test were used to compare the outcome of both studies (2-sided P values) assuming no difference in population or sensitivity of the parasite strain. The 2-tailed paired *t*-test and the Kruskal-Wallis tests were employed to compare the clinical parameters before and after treatment.

## Results

### Baseline Characteristics

Of 584 villagers and 51 school-aged children screened in El-Haddad El-Bahary village (study 1), 22 individuals were found *Fasciola*-positive. Two patients were excluded (pregnancy, n = 1; age below 5 years, n = 1). Twenty patients (10 females, 10 males; aged 5–70 years with a mean of 24 years) were included in study 1 (Table 1).

**Table 1:** Demographic baseline characteristics of 57 *Fasciola-*infected patients at inclusion.

	Treatment			
Characteristics	Artemether(6×80 mg)	Artemether(3×200 mg)	Triclabendazole(10 mg/kg)	Triclabendazole(20 mg/kg)
No. of patients treated	20	17	16	4
Males/females	10/10	7/10	6/10	2/2
Mean (SD) age, years	24.4 (21.5)	13.9 (5.8)	14.4 (5.8)	16.3 (2.5)
Mean (SD) weight, kg	43.9 (23.1)	49.5 (24.4)	55.4 (26.2)	66.3 (19.8)
Range of actual total dose (mean), mg/kg	6–26.7 (14.4)	8.3–31.6 (15.8)	n.d.	n.d.

n.d., not determined; SD, standard deviation.

In the second study, 631 individuals were examined and 19 *Fasciola*-positive subjects were identified. Of these, 17 patients (10 females, 7 males; aged 5–26 years, with a mean of 14 years) (Table 1) were included in the study. However, two of the positive cases were excluded because the initial diagnosis by the Ministry of Health and Population could not been confirmed.

The baseline GM *Fasciola* FECs in the two studies were 28.3 EPG and 29.1 EPG (Table 2). Twenty-six individuals were classified as lightly infected (1–99 EPG), whereas 10 individuals had a moderate/heavy infection (≥100 EPG). Ten participants were concurrently infected with *Fasciola* spp. and *S. mansoni*, and one patient was identified with a double infection of *Fasciola* spp. and *Hymenolepis nana*.

**Table 2:** Effect of artemether administered at two different regimens to patients infected with *Fasciola* spp.

Treatment	*Fasciola* spp.						*S. mansoni*		
	Infection intensity	No. of patients treated	No. of patients cured (%)	Pre-treatment GM *Fasciola* egg count (EPG)	Post-treatment GM *Fasciola* egg count (EPG)	ERR (%)	No. of patients treated	No. of patients cured (%)	ERR (%)
Study 1(6×80 mg artemether)	All infections	20*	7 (35)**	28.3	10.4	63**	5	3 (60)	59
	1–99 EPG	13	7 (54)**	12.6	4.2	67			
	≥100 EPG	6	0	161.0	72.7	55			
Study 2(3×200 mg artemether)	All infections	17	1 (6)	29.1	32.0	0	5	3 (60)	n.d.
	1–99 EPG	13	1 (8)	18.8	21.7	0			
	≥100 EPG	4	0	119.6	113.0	5.5			
Follow-up: triclabendazole (10 mg/kg)		16	11 (69)**	32.0	2.0	94***			
Follow-up: triclabendazole (20 mg/kg, split dose)		4	3 (75)	39.6	1.5	96			

EPG, eggs per gram of stool; ERR, egg reduction rate; GM, geometric mean; n.d., not determined.

*One patient was diagnosed positive with MIF only, hence no quantitative egg count is available at baseline.

**CR significantly different (P<0.05) from study 2 (3×200 mg artemether).

***ERR highly significantly different (P<0.001) from study 2 (3×200 mg artemether).

### Efficacy of Artemether

**Figure 1:**
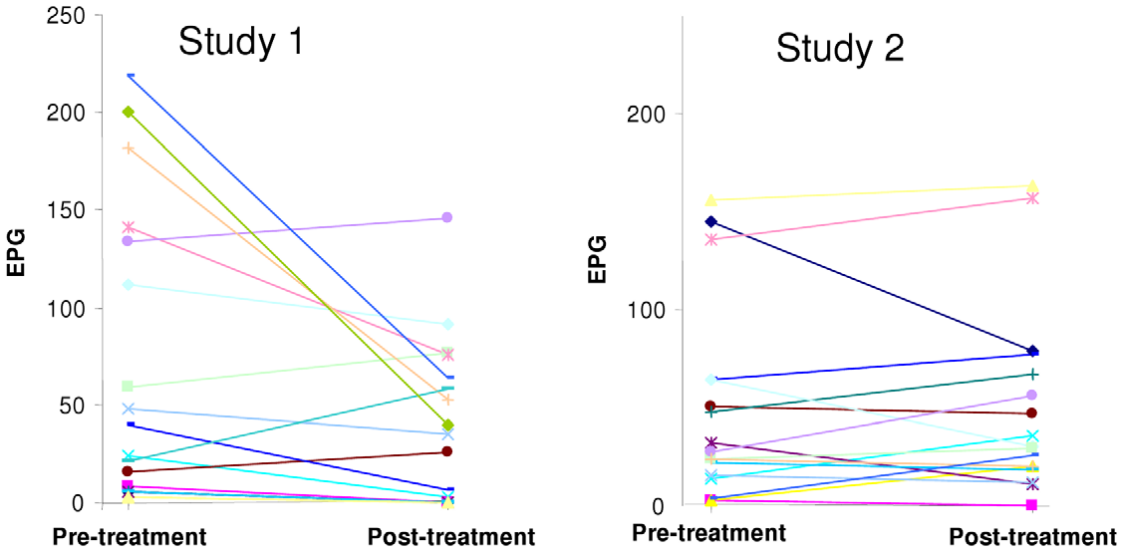
Pretreatment and posttreatment *Fasciola* egg counts in patients following two artemether regimens. Study was carried out in Egypt and *Fasciola*-infected individuals were treated with either 6×80 mg artemether (study 1) or 3×200 mg artemether (study 2).

Data from all patients were included in the analysis, as no patient was lost to follow-up (per-protocol analysis). CRs achieved with 6×80 mg and 3×200 mg artemether were 35% and 6%, respectively (Table 2). Fisher's exact test showed a statistical difference between the CRs obtained with the different treatment schedules (P = 0.048; 95% CI: 0.002–1.15). None of the patients characterized by an infection intensity of 100 EPG and above was cured after artemether administration regardless of the treatment regimen, while CRs documented in patients with a light *Fasciola* infection were 54% (6×80 mg artemether) and 8% (3×200 mg artemether) (CRs of light infections were significantly higher in study 1 compared to study 2; P = 0.013; 95% CI: 0.001–0.77). Treatment with artemether over 3 consecutive days resulted in ERRs of 63% (67% for light infections and 55% for infections ≥100 EPG). The individual pretreatment and posttreatment FECs are presented in Figure 1. No effect on FECs were observed when artemether was administered on a single treatment day with the exception of a very low ERR of 6% among patients with an infection intensity ≥100 EPG. The overall ERR between the two studies differed significantly (P<0.001).

In each of the two studies, five patients were co-infected with *S. mansoni*. At treatment follow-up, three out of the five patients in each study were recorded egg-free (CR: 60%).

### Efficacy of Triclabendazole

Sixteen patients who were still found *Fasciola*-positive after treatment with 3×200 mg artemether were administered a single 10 mg/kg oral dose of triclabendazole. CR and ERR were 69% and 94%, respectively; significantly higher than CR (P<0.001; 95% CI: 3.19–1605.7) and ERR (P<0.001) observed following treatment with 3×200 mg artemether. The infection intensity did not influence the treatment outcome (data not shown). Four out of five patients who were still passing *Fasciola* eggs following a single triclabendazole dose were provided a double dose of triclabendazole and the respective CR and ERR were 75% and 96%.

### Safety Assessment

#### Clinical chemistry variables

There were no noteworthy effects of artemether on the liver enzymes and renal function parameters, with the exception of a statistically significant increase in GGT 5 days after the final dosing of artemether (6×80 mg) (Table 3). Following treatment with 3×200 mg artemether, GGT values were lower 28 days posttreatment when compared to baseline values. ALT values significantly decreased between the first and second follow-up time point. Finally, the values for ALP were above the reference range before and after treatment with artemether given over 3 consecutive days. Hematological parameters were not found to significantly differ from baseline values, with the exception of hemoglobin, which was significantly increased 28 days posttreatment with 6×80 mg artemether.

**Table 3:** Liver and renal function and hematological parameters pre- and posttreatment with artemether.

Parameter	Reference range	Study 1 (6×80 mg artemether)			Study 2 (3×200 mg artemether)		
		Day of analysis			Day of analysis		
		Pretreatment day 0 (mean ± SD)	Post-treatment day 7 (mean ± SD)	Post-treatment day 28 (mean ± SD)	Pretreatment day 0 (mean ± SD)	Posttreatment day 5 (mean ± SD)	Posttreatment day 28 (mean ± SD)
Alkaline phosphatase (ALP) (IU/L)	30–120	162.1 (17.3)	163.4 (16.1)	161.1 (17.1)	65.8 (30.2)	63.8 (28.8)	61.3 (29.8)
Alanine transaminase (ALT) (IU/L)	9–60	4.8 (0.6)	4.1 (0.4)	5.2 (0.5)	3.4 (4.6)	3.2 (2.2)	2.0 (1.0)**
Aspartate transaminase (AST) (IU/L)	10–40	10.0 (0.3)	10.2 (0.4)	13.2 (2.1)	3.4 (2.7)	3.8 (4.3)	3.3 (2.5)
Gamma glutamyl transpeptidase (GGT) (IU/L)	0–51	10.7 (1.3)	12.2 (1.3)*	10.8 (1.4)**	8.0 (2.4)	7.3 (1.7)	6.4 (2.3)*
Bilirubin (mg/dl)	0.2–1.2	0.4 (0.1)	0.4 (0.1)	0.3 (0.03)	0.4 (0.1)	0.4 (0.1)	0.4 (0.1)
Urea (mg/dl)	12–48	29.0 (2.0)	29.7 (1.3)	28.8 (1.1)	35.2 (5.6)	35.2 (5.6)	34.6 (6.0)
Creatinine (mg/dl)	0.6–1.3	0.7 (0.03)	0.8 (0.04)	0.7 (0.03)	0.9 (0.3)	0.9 (0.2)	0.8 (0.1)
Eosinophils (%)	0–6	3.3 (0.4)	4.0 (0.4)	3.4 (0.4)	3.4 (1.9)	4.3 (2.8)	4.3 (2.8)
Hemoglobin (g/dl)	11.5–17.5	10.4 (0.3)	10.45 (0.3)	11.17 (0.2)*,**	13.6 (1.2)	13.4 (1.4)	13.0 (1.1)
Total leucocytes (mm^3^)	3900–10,000	6300 (179)	6253 (181)	6028 (195)	6010 (976)	6055 (1110)	6421 (874)

*Statistically significant difference from baseline (p<0.05).

**Statistically significant difference between first and second follow-up survey.

The comparison between pre- and posttreatment values of liver and renal function and hematological parameters showed no significant differences following administration of triclabendazole (10 and 20 mg/kg) (Table 4) apart from slight variations in bilirubin and hemoglobin levels, which were slightly lower 7 days posttreatment, compared to baseline and the second follow-up 28 days posttreatment.

**Table 4:** Liver and renal function and hematological parameters pre- and posttreatment with triclabendazole.

Parameter	Reference range	10 mg/kg triclabendazole (n = 16)			20 mg/kg triclabendazole (n = 4)		
		Day of analysis			Day of analysis		
		Pretreatment day 0 (mean ± SD)	Post-treatment day 5 (mean ± SD)	Post-treatment day 28 (mean ± SD)	Pretreatment day 0 (mean ± SD)	Post-treatment day 5 (mean ± SD)	Post-treatment day 28 (mean ± SD)
Alkaline phosphatase (ALP) (IU/l)	30–120	133.7 (38.8)	137.3 (39.9)	135.8 (37.7)	111.0 (65.1)	106.4 (59.2)	99.5 (32.2)
Alanine transaminase (ALT) (IU/l)	9–60	10.9 (2.3)	16.0 (11.4)	11.9 (2.5)	18.3 (3.2)	19.3 (8.1)	20.5 (9.3)
Aspartate transaminase (AST) (IU/l)	10–40	10.6 (3.9)	19.6 (23.2)	13.1 (3.0)	23.5 (10.4)	29.3 (13.7)	29.3 (8.7)
Gamma glutamyl transpeptidase (GGT) (IU/l)	0–51	7.0 (1.4)	8.3 (2.6)	7.7 (1.8)	7.2 (1.1)	9.4 (3.3)	9.4 (3.3)
Bilirubin (mg/dl)	0.2–1.2	0.6 (0.2)	0.4 (0.3)*	0.6 (0.2)**	0.4 (0.1)	0.6 (0.3)	0.6 (0.1)
Urea (mg/dl)	12–48	37.9 (11–0)	35.2 (12.6)	34.7 (6.6)	23.8 (2.0)	18.1 (6.3)	22.2 (2.7)
Creatinine (mg/dl)	0.6–1.3	0.8 (0.2)	0.7 (0.2)	0.8 (0.1)	0.9 (0.3)	1.1 (0.4)	0.9 (0.2)
Eosinophils (%)	0–6	4.5 (2.5)	3.3 (1.5)	3.5 (1.2)	3.8 (1.0)	3.5 (1.0)	4.0 (0.8)
Hemoglobin (g/dl)	11.5–17.5	12.8 (1.8)	11.3 (1.1)*	12.5 (1.3)**	13.5 (0.6)	13.3 (0.8)	12.9 (0.6)
Total leucocytes (mm^3^)	3900–10,000	6356 (951)	5950 (1074)	6707 (918)	5300 (902)	5900 (1320)	6350 (661)

*Statistically significant difference from baseline (p<0.05).

**Statistically significant difference between first and second follow-up survey.

#### Adverse events

Both artemether regimens were well tolerated and no participant required special medical follow-up. As summarized in Table 5, adverse events included abdominal pain, fatigue, headache, vomiting, and diarrhea. Overall, 42 mild and two moderate episodes of adverse events were reported when artemether was given on 3 consecutive days. A slightly higher number of adverse events was documented (n = 58) in patients receiving artemether on a single day. However, all of these were mild. The frequency of adverse events was similar among the two treatment regimens, with the exception of headache and fever, which were more commonly reported in the second study (single treatment day). Importantly though, adverse events were also present prior to treatment and some of them occurred only 96 h posttreatment, suggesting that they might not have been treatment-related.

**Table 5:** Treatment related adverse events observed in patients receiving artemether.

Treatment	Adverse event	Grading	No. of adverse events (%)				
Study 1 (6×80 mg artemether) (n = 20)			**Examination point (hours post-treatment)**				
			**24**	**48**	**72**	**96**	**120**
	Abdominal pain	Mild	0	6 (30)	1 (5)	7 (35)	5 (25)
		Moderate	0	1 (5)	0	0	0
	Fatigue	Mild	0	4 (20)	1 (5)	1 (5)	0
		Moderate	0	0	0	0	0
	Headache	Mild	0	1 (5)	1 (5)	3 (15)	0
		Moderate	0	0	0	0	0
	Diarrhea	Mild	0	0	5 (25)	5 (25)	0
		Moderate	0	1 (5)	0	0	0
	Nausea	Mild	0	0	0	0	0
		Moderate	0	0	0	0	0
	Vomiting	Mild	1 (5)	1 (5)	0	0	0
		Moderate	0	0	0	0	0
Study 2 (3×200 mg artemether) (n = 17)			**Morning**	**Mid day**	**Evening**	**96 h**	
	Abdominal pain	Mild	1 (5)	1 (5)	4 (21)	6 (32)	
	Fatigue	Mild	0	0	0	4 (21)	
	Headache	Mild	3 (16)	0	3 (16)	13 (69)	
	Diarrhea	Mild	0	0	1 (5)	2 (11)	
	Nausea	Mild	0	0	0	3 (16)	
	Vomiting	Mild	0	0	0	4 (21)	
	Fever	Mild	0	0	0	6 (32)	
	Dizziness	Mild	3 (16)	0	3 (16)	1 (5)	

Abdominal pain was more often observed after treatment with triclabendazole (Table 6) than after artemether regimens. Headache and dizziness were other commonly observed adverse events. However, many of the symptoms might have been disease- rather than treatment-related, as they were already reported before drug administration.

**Table 6:** Treatment-related adverse events observed in patients receiving triclabendazole.

Treatment	Adverse event	Grading	No. of adverse events (%)		
			Examination point (hours posttreatment)		
			24	48	72
Triclabendazole (10 mg/kg) (n = 16)	Abdominal pain	Mild	0	8 (50)	8 (50)
		Moderate	0	1 (6.3)	3 (18.8)
	Fatigue	Mild	0	0	1 (6.3)
		Moderate	0	0	0
	Headache	Mild	0	5 (31.3)	5 (31.3)
		Moderate	0	0	1 (6.3)
	Diarrhea	Mild	0	0	4 (25)
		Moderate	0	0	1 (6.3)
	Nausea	Mild	0	0	0
		Moderate	0	0	0
	Vomiting	Mild	0	2 (12.5)	2 (12.5)
		Moderate	0	0	0
	Fever	Mild	0	0	1 (6.3)
		Moderate	0	2 (12.5)	0
	Dizziness	Mild	0	10 (62.5)	4 (25)
		Moderate	0	0	0
Triclabendazole (20 mg/kg) (n = 4)	Abdominal pain	Mild	0	4	2
		Moderate	0	0	0
	Fatigue	Mild	0	2 (50)	0
		Moderate	0	0	0
	Headache	Mild	3 (75)	2 (50)	1 (25)
		Moderate	0	0	1 (25)
	Diarrhea	Mild	0	1 (25)	1 (25)
		Moderate	0	0	0
	Nausea	Mild	1 (25)	3 (75)	0
		Moderate	0	0	0
	Vomiting	Mild	0	1 (25)	1 (25)
		Moderate	0	0	0
	Fever	Mild	1 (25)	1 (25)	0
		Moderate	0	0	1 (25)
	Dizziness	Mild	2 (50)	2 (50)	0
		Moderate	0	0	0

## Discussion

While the veterinary importance of fascioliasis cannot be overemphasized, this zoonotic disease is also of considerable and growing public health importance, yet it often remains neglected. A major challenge is that treatment is restricted to a single drug, i.e., triclabendazole, which is registered for human use only in Ecuador, Egypt, France, and Venezuela [7]. Results from a study carried out in Vietnam raised some hope for an alternative; artesunate administered to patients with symptomatic fascioliasis pointed to a potential role of the artemisinins against fascioliasis. Indeed, the authors concluded that it is worthwhile to investigate this drug class in more detail, including additional clinical trials [20]. We now present the first results with artemether in the treatment of chronic fascioliasis in two epidemiological settings of Egypt. Artemether (monotherapy) was administered following the dosing regimen of a commonly used ACT, the 6-dose regimen of artemether-lumefantrine [21], and a previously employed 3-dose malaria treatment schedule administered on a single day [22]. Egypt was selected because of the known fascioliasis endemicity, particularly in the Nile Delta, and the absence of malaria [28], [29]. The prevalence of *Fasciola* spp. observed in the two study sites (i.e., Behera and Alexandria; prevalence 3–4%) was similar to previous studies in these areas [5], [28], [30], despite frequent community treatment programs with triclabendazole.

Our study failed to extend promising findings obtained with the artemisinins in rats experimentally, and sheep naturally, infected with *F. hepatica* [31]. Indeed, we found low CRs (6–35%) when artemether was given at two different malaria treatment schedules. Nonetheless, a moderate ERR of 63% was observed following the 6-dose course of artemether. The difference in the ERR between the two artemether treatment schedules (nil *vs.* 63%) is striking, yet difficult to explain. Since the half life of artemether is very short (<1 h) [32], parasite exposure to the drug might have been insufficient if the drug is given on a single treatment day. However, detailed *in vitro* drug sensitivity and pharmacokinetic studies are required to further elucidate this issue. It is interesting to note that the CRs (nil *vs.* 54%) and ERRs (55% *vs.* 67%) were higher in patients classified as lightly infected compared to moderate/heavy infections in the 6-dose regimen. A similar trend was observed in a recent study, which assessed the efficacy of an artesunate-sulfalene plus pyrimethamine combination in *S. mansoni*-infected school-aged children in Kenya: significantly higher CRs were observed in children harboring a light *S. mansoni* infection compared to moderate and heavy infections [33].

In the present study, five participants in each of the two studies were co-infected with *Fasciola* spp. and *S. mansoni*. Moderate CRs were observed against *S. mansoni* (60%) regardless of the selected treatment regimen. This finding is in line with previous studies, which documented low-to-moderate efficacies of an artemisinin monotherapy in the treatment of chronic infections with *Schistosoma* spp. [34]–[36]. An opposite trend, a CR of 70% and an ERR of 86% was reported following treatment of Nigerian children using two doses of artesunate at 6 mg/kg given 2 weeks apart [37]. In recent years also the effect of ACTs on schistosomiasis has been studied (for a summary of studies, see Utzinger et al. (2010) [38]) Overall, a moderate efficacy was observed using ACTs against the two major schistosome species, *S. mansoni* and *S. haematobium*. Although promising results were obtained in small exploratory trials with the artemisinins against schistosomiasis, larger clinical trials could not confirm these findings, and hence praziquantel remains the drug of choice [33], [38], [39].

CRs of 69% and 75% were observed in patients treated with triclabendazole at 10 mg/kg and 20 mg/kg, respectively. The observed efficacy is slightly lower than a calculated overall CR of 83% following 10 mg/kg and reported CRs ranging from 93 to 100% following a double dose of triclabendazole [40]. Additionally, a recent study with 10 mg/kg triclabendazole in Egypt reported a complete cure following triclabendazole (10 mg/kg) [41]. However, care is indicated in these comparisons because of the small sample sizes in the current study, although strain differences in the susceptibility of *Fasciola* spp. to triclabendazole might play a role in the somewhat lower efficacies observed here compared to previous studies. Participants treated with triclabendazole showed a higher incidence of abdominal pain compared to those treated with artemether, which might be related to the higher efficacy of triclabendazole (dying worms).

In conclusion, significantly higher CRs and ERRs were observed with triclabendazole when compared to artemether, the latter administered following two malaria treatment schedules. Hence, triclabendazole remains the drug of choice against fascioliasis. In view of threatening triclabendazole resistance development, concerted efforts are required, including structure-activity relationships with the synthetic peroxides in *F. hepatica*-infected rats [42]. Combination chemotherapy is also recognized as a potential strategy for reducing the emergence of drug resistance [43], [44]. Since we have observed synergistic interactions of combinations of triclabendazole (2.5 mg/kg) plus artemether (6.25–100 mg/kg) on adult worm burden in *F. hepatica*-infected rats [15] further preclinical studies to investigate the efficacy and safety of an artemether-triclabendazole combination are warranted. Combination chemotherapy with artemether and triclabendazole might offer an advantage over triclabendazole monotherapy, in particular in the case of possible future treatment failures with triclabendazole alone.

## Supporting Information

Checklist S1 CONSORT Checklist.
(DOC)

Protocol S1 Trial Protocol.
(DOC)
