# Supplementary material for: Efficacy and Safety of Artemether in the Treatment of Chronic Fascioliasis in Egypt: Exploratory Phase-2 Trials
Source: PLoS Negl Trop Dis. 2011 Sep 6;5(9):e1285. doi: 10.1371/journal.pntd.0001285 (PMC3167773; doi:10.1371/journal.pntd.0001285)
Supplement: Protocol S1 — Trial Protocol. (DOC) [file pntd.0001285.s002.doc]

**Research protocol**

Theodor Bilharz Institute and Swiss Tropical Institute (STI)

**Title**

**Safety and efficacy of oral artemether against *Fasciola spp.* infections in Egyptian patients**

**Appendices**

**Appendix 1:** Personal data and clinical examination form

**Appendix 2:** Summary and grading of adverse events

**Investigators**

**Prof. Dr. Sanaa Botros (Pharmacologist; Co-PI)**

Theodor Bilharz Research Institute

Department of Pharmacology

P.O.30 Imbaba, Giza, Egypt

Tel.: 202-540-1019 (extension 160)

Fax:202-540-8125

E-mail: sanaabotros@link.net>

**Prof. Hoda Sabry (Parasitologist; Co-investigator)**

Theodor Bilharz Research Institute

Department of Parasitology

P.O.30 Imbaba, Giza, Egypt

Tel.: 202-540-1019 (extension 196)

Fax: 202-540-8125

E-mail: dr_hodasabry@hotmail.com

**Prof. Maged El-Ghanam (Physician; Co-investigator)**

Theodor Bilharz Research Institute

Department of Hepatology, gastroenterology and tropical medicine

P.O.30 Imbaba, Giza, Egypt

Tel.: 202-540-1019 (extension 257&258)

Fax: 202-540-8125

E-mail:elghannam@yahoo.com

**Prof. Hanan Sayed (Public health specialist; Co-investigator)**

Theodor Bilharz Research Institute

Department of Public Health

P.O.30 Imbaba, Giza, Egypt

Tel.: 202-540-1019 (extension 222)

Fax: 202-540-8125

E-mail: hananasayed@hotmail.com

**Prof. Jennifer Keiser (Pharmacologist; Co-PI)**

Swiss Tropical Institute

Socinstr. 57

CH- 4002 Basel, Switzerland

Tel.: +41 61 284-8218

Fax: +41 61 284-8105

E-mail: [jennifer.keiser@unibas.ch](mailto:jennifer.keiser@unibas.ch)

**Prof. Christoph Hatz (Physician; Co-investigator)**

Swiss Tropical Institute

Socinstr. 57

CH- 4002 Basel, Switzerland

Tel.: +41 61 284-8255

Fax: +41 61 284-8283

E-mail: christoph.hatz@unibas.ch

**Prof. Jürg Utzinger (Epidemiologist; Co-investigator)**

Department of Public Health and Epidemiology

Swiss Tropical Institute

Socinstr. 57

CH- 4002 Basel, Switzerland

Tel.: +41 61 284-8129

Fax: +41 61 284-8105

E-mail: juerg.utzinger@unibas.ch

**Prof. Marcel Tanner (Epidemiologist and Director; Co-investigator)**

Swiss Tropical Institute

Socinstr. 57

CH- 4002 Basel, Switzerland

Tel.: +41 61 284-8283

Fax: +41 61 284-8105

E-mail: marcel.tanner@unibas.ch

**1. Background**

Fascioliasis is a disease caused by the infection of liver flukes of the species *Fasciola hepatica* and *F. gigantica*. It is a zoonotic disease that is of great veterinary importance and considerable public health significance worldwide. In humans, an infection with *Fasciola* spp. occurs through oral ingestion of metacercariae either through consumption of raw, undercooked or unwashed aquatic plants, or through drinking contaminated water (Mas-Coma, 2004). Chronic infections can lead to severe disabling diseases, including hepatic lesions, fibrosis and chronic inflammation of the bile ducts (Richter et al., 2002; Mas-Coma, 2004). An estimated 91 million people are at risk of fascioliasis mainly in the Andean countries of South America, Cuba, Western Europe, Egypt and the Islamic Republic of Iran (Keiser and Utzinger, 2005). It is estimated that between 2.4 and 17 million people are currently infected with *F. hepatica* and/or *F. gigantica* (WHO, 1995; Esteban et al., 2003). In some parts of Egypt human fascioliasis has become an important clinical problem and prevalence rates of 13-17% have been reported (Hammouda et al., 1995). High *F. hepatica/F. gigantica* prevalences have been reported in school aged children (Esteban et al., 2003).

There is no fascioliasis vaccine available and hence chemotherapy is the current mainstay for morbidity control of the disease. However, chemotherapy of fascioliasis relies on a single drug, namely triclabendazole (Egaten®) (Keiser et al., 2005). Although, triclabendazole has been routinely used for more than 2 decades in veterinary medicine, it is currently registered in only 4 countries to treat patients infected with *Fasciola* spp. (Keiser et al., 2005). Bithionol, emetine and dehydroemetine are still used for the treatment of fascioliasis, but all of these agents can cause serious adverse events (Chen and Mott, 1990; Apt et al., 1995).

Against this background, there is a pressing need for discovery and development of novel fasciocidal drugs. This is justified on the following three grounds. First, in the absence of a vaccine, and the challenges surrounding implementation of preventive measures such as access to clean water and improved sanitation and the difficulties in changing human behavior (i.e. thoroughly washing and cooking aquatic plants) control of food-borne trematodiasis in general and fascioliasis in particular rests on morbidity control. However, as mentioned above chemotherapy of fascioliasis relies on triclabendazole alone. Second, the dependence on a single drug for treatment and control of fascioliasis is an alarming situation, particularly in view of mounting evidence of drug resistance development. Triclabendazole resistance is already common in sheep and cattle (Keiser et al., 2005). Third, the development and delivery of new drugs – singly or in combination with drugs that have a different mechanism of action – is a key factor in enhancing therapeutic outcomes and prolonging the useful life-span of triclabendazole (White, 1999; Olliaro and Taylor, 2004).

In our previous study carried out between April and July 2007 in Egypt we found that a 3 day dose schedule of 80 mg artemether twice daily resulted in a cure rate of 35%. We would now like to follow up on this promising result and investigate the effect of slightly higher doses of artemether, namely 600 mg artemether in 3 divided doses 6 h apart. This treatment regimen has been used safely in the treatment of malaria (Nosten, 1994).

**2. Detailed research plan**

**2.1. Study aims**

To assess the safety and efficacy of oral artemether in Egyptian patients infected with *F. hepatica/ F. gigantica*

**3.2. Experimental design**

*Study area and patients*

The study will be carried out as a proof of concept open label trial in approximately 20 patients based on guidelines by Julius et al at a governorate of lower Egypt and will include a maximum of 18 patients (15 patients and an additional 3 to compensate for patients leaving the study voluntarily) with a parasitological-confirmed infection with *F. hepatica* and/or *F. gigantica*. We will include children (11 years and older) as *F. hepatica/F. gigantica* prevalences and morbidity are high in school age children.

*Patient inclusion criteria*

1.) Age 11-70 years, both sexes

2.) For married females, not pregnant, as assessed by the medical doctor last menstrual cycle, upon initial clinical assessment

3.) Absence of major systemic illnesses, as assessed by the medical doctor, upon initial clinical assessment

4.) Infection with *F. hepatica* and/or *F. gigantica* as confirmed by standard parasitological stool examination

5.) No known or reported hypersensitivity to artemether

6.) No known or reported history of chronical illness as cancer, diabetes, hypertension, chronic heart, liver or renal disease.

7.) Full clinical examination

8.) Written informed consent

*Patient exclusion criteria*

1.) Presence of any abnormal medical condition, judged by the medical doctor. If several patients experience serious adverse events the study will be stopped

2.) Severe liver disease of other etiology

3.) Recent history of anthelminthic drugs (triclabendazole, albendazole, bithionol, dehydroemetine, praziquantel within past 4 weeks)

4.) Attending other clinical trials during the study

5.) For females: pregnancy, lactation

Patient who were found with a *F. hepatica*/*F. gigantica* infection, but who were excluded from the study due to one or several of the above mentioned exclusion criteria will undergo a complete medical examination and will be provided a single oral dose of 10 mg/kg triclabendazole (Egaten®) and if necessary spasmolytic drugs according to standard procedures of the Ministry of Health and Population in Egypt.

*Baseline survey*

Medical history of patients participating in the study will be assessed with a standardized questionnaire, in addition to full clinical examination carried out by clinician. Blood samples will be collected for complete haemogram, including sedimentation rate, red, white and differential blood cell count, liver (alkaline phosphatase, alanine amino transferase, gamma glutamyltransferase, bilirubin) and kidney functions (serum urea and creatinine) as well as for diagnostic tests, which will be carried out in Egypt and Switzerland.

*F. hepatica* and/or *F. gigantica* infection intensity: Three stool samples of patients collected within 5 consecutive days will be examined utilizing the Kato Katz method Flotac, and the Formol-ether sedimentation technique simultaneously. A total of 9 Kato-Katz thick smears will be prepared (3 smears from each of the 3 stool specimen). Infection intensity (arithmetic mean egg count per gram of stool) will be calculated.

*Treatment*

As malaria is not present in the study area we will use an artemether monotherapy treatment regimen.

Artemether capsules (Artemidine®--50 mg/tablets) will be provided by Kunming pharmaceuticals. Artemidine® is manufactured according to international Good Manufacturing Practice (GMP) standards and fully licensed for human use as antimalarial treatment.

We will evaluate the effectiveness of oral artemether administered to 18 patients (15 patients and an additional 3 to compensate for patients leaving the study voluntarily) as a 3 dose treatment regimen closely mirroring a previously commonly used antimalarial treatment regimen: i.e. a three dose regimen with 600 mg artemether in 3 divided doses in intervals of 6 hours. This artemether treatment schedule has been used safely in a large number of patients (Nosten, 1994).

A spasmolytic drug will be administered one day before treatment with artemether and once daily within the period of artemether treatment.

All tablets will be administered in the presence of the investigator(s), and ingestion confirmed. This will be recorded with the time and date of closing. Subjects will be asked not to take any drugs other than those prescribed by the study medical team. After ingestion of artemether, the subjects will be observed for 1 hour to ensure retention of the drug. Vomiting within 1-hour post dosing will require re-dosing. The subjects will not allow more than one repeated dose. No re-administration will be needed for subjects vomiting after 1 hour.

*Morbidity monitoring*

Patients receiving artemether will be visited daily by the clinician during the period of drug administration. They will be kept for observation for at least 3 hours for any acute adverse events (AEs). If there is any abnormal finding, the study physicians will perform full physical examination on each individual and findings will be recorded. AEs within 24 hours of drug administration will be assessed. Those patients who report AEs will be examined carefully by the study physician and, when necessary, action will be taken (see appendix). Details of adverse experiences will be recorded by the medical doctors during the trial, including variables describing their incidence, onset, cessation, duration, intensity, frequency, seriousness, and causality (as determined by the physician), as well as any action taken (for grading of adverse events see appendix).

Follow-up after treatment will be on day 2 and 3 and 4-5 weeks later. In addition to clinical examination during follow-up periods, blood samples will be collected for complete haemogram, including sedimentation rate, red, white and differential blood cell count, liver (alkaline phosphatase, alanine amino transferase, gamma glutamyltransferase, bilirubin) and kidney functions (serum urea and creatinine).

Patients suffering from acute fasciolosis will be immediately treated with 10 mg/kg triclabendazole (Egaten®, Novartis) and spasmolytic drugs and excluded from the study.

Intensity of adverse events will be judged by the medial doctor as followed:

*Mild:* present but not required any interference.

*Moderate:* 1) present and required medication for symptomatic relief as requested by the affected people or 2) present and interfering with normal daily activities.

*Severe:* present and required medical intervention beyond symptomatic relief

Relationship of adverse events to the study medication will be determined. The probability of relation with treatment will be graded by the medical doctor. Changes in clinical signs and symptoms related to fascioliasis (nausea, abdominal pain, diarrhea, urticaria and pruritus) will be monitored.

*Endpoint parasitological stool survey and clinical evaluation*

*F. hepatica and/or F. gigantica*  infection intensity: 28 days after the last artemether dose has been administered patients stool samples will again be examined for the number of *F. hepatica* *and/or F. gigantica*  eggs in 3 different stool samples. Participants will be considered *F. hepatica* negative if no eggs have been found in 3 stool specimens analyzed by 3 Kato-Katz thick smears and Formol-Ether sedimentation technique taken from the same stool sample from each patient. Infection intensity (arithmetic mean egg count per gram of stool) will be calculated.

All patients will be treated with 10 mg/kg triclabendazole (Egaten®).

At the end point clinical evaluation blood samples will be collected for complete haemogram, including sedimentation rate, red, white and differential blood cell count, liver (alkaline phosphatase, alanine amino transferase, gamma glutamyltransferase, bilirubin) and kidney functions (serum urea and creatinine).

*Concomitant therapy*

All medications taken one month before and during the study period must be recorded with indication, dose regimen, date and time of administration.

### Medication(s)/treatment(s) permitted during the trial

- Analgesics and antipyretics are allowed to be given to the subjects in case of fever, antiemetics to prevent nausea and vomiting, spasmolytics to reduce the symptoms associated with parasite expulsion and/or antibiotics to prevent or treat bacterial superinfection.

### Medication(s)/treatment(s) NOT permitted during the trial

- - No other active drugs against worms such as Triclabendazole, Praziquantel, Albendazole, Dehydroemetine, Emetine, Bithionol within 4 weeks are permitted during the trial

**3. References**

Apt, W., X. Aguilera, F. Vega, C. Miranda, I. Zulantay, C. Perez, M. Gabor, and P. Apt. 1995. Treatment of human chronic fascioliasis with triclabendazole: drug efficacy and serologic response. American Journal of Tropical Medicine and Hygiene 52: 532-535.

Chen, M. G., and K. Mott. 1990. Progress in assessment of morbidity due to *Fasciola hepatica* infection: a review of recent literature. Tropical Dieseases Bulletin 87: 1-38.

Esteban, J.-G., C. Gonzalez, F. Curtale, C. Muñoz-Antoli, M. A. Valero, M. D. Bargues, M. el-Sayed, A. A. W. el-Wakeel, Y. Abdel-Wahab, A. Montresor, D. Engels, L. Savioli, and S. Mas-Coma. 2003. Hyperendemic fascioliasis associated with schistosomiasis in villages in the Nile Delta of Egypt. American Journal of Tropical Medicine and Hygiene 69: 429-437.

Hammouda, N. A., S. T. el-Mansoury, M. Z. el-Azzouni, and Y. el-Gohari. 1995. Therapeutic effect of triclabendazole in patients with fascioliasis in Egypt. A preliminary study. Journal of the Egyptian Society of Parasitology 25: 137-143.

Keiser, J., D. Engels, G. Büscher, and J. Utzinger. 2005. Triclabendazole for the treatment of fascioliasis and paragonimiasis. Expert Opinion of Investigational Drugs 14: 1513-1526.

Keiser, J., and J. Utzinger. 2005. Emerging foodborne trematodiasis. Emerging Infectious Diseases 11: 1507-1514.

Mas-Coma, S. 2004. Human fascioliasis: epidemiological patterns in human endemic areas of South America, Africa and Asia. Southeast Asian Journal of Tropical Medicine and Public Health 35: 1-11.

Nosten, F. 1994. Artemisinins: large community studies. Transactions of the Royal Society of Tropical Medicine and Hygiene 88: 45-46.

Olliaro, P. L., and W. R. Taylor. 2004. Developing artemisinin based drug combinations for the treatment of drug resistant falciparum malaria: A review. Journal of Postgraduate Medicine 50: 40-44.

Price, RN, Nosten, F, Luxemburger, C. , Kham, A., Brockman A., Chongsuphajaisiddhi T., White, NJ. 1995 Artesunate versus artemether in combination with mefloquine for the treatment of multidrug-resistant falciparum malaria. Transactions of the Royal Society of Tropical Medicine and Hygiene 89:523-527.

Richter, J., M. Knipper, K. Goebels, and D. Haeussinger. 2002. Fascioliasis. Current Treatment Options in Infectious Diseases 4: 313-317.

White, N. 1999. Antimalarial drug resistance and combination chemotherapy. Philosophical transactions of the Royal Society of London. Series B, Biological sciences. 354: 739-749.

WHO. 1995. Control of foodborne trematode infections. Report of a WHO study group. WHO Tech Rep Ser No. 849. World Health Organization.Geneva.

**Appendix 1: Personal data and clinical examination form**

Date : _____/______/ 2007 (jj/mm)

| Name |  |
| --- | --- |
| First name |  |
| Address |  |
|  |  |
| Date of birth |  |

| Temperature | °C |
| --- | --- |
| Weight | kg |
| Height | cm |
| Blood pressure | # / # mm Hg |
| Pulse | # / min |

| **History** |  |
| --- | --- |
| History of hypertension | 🞎 yes 🞎 no |
| History of diabetes | 🞎 yes 🞎 no |
| History of chronic illnesses | 🞎 yes 🞎 no |

| **Clinical picture** |  |
| --- | --- |
| Intermittent fever | 🞎 yes 🞎 no |
| Headache | 🞎 yes 🞎 no |
| Nausea | 🞎 yes 🞎 no |
| Vomiting* | 🞎 yes 🞎 no |
| Diarrhea | 🞎 yes 🞎 no |
| Dull Abdominal pain | 🞎 yes 🞎 no |
| Collical pain | 🞎 yes 🞎 no |
| Vertigo | 🞎 yes 🞎 no |
| Malaise and weight loss | 🞎 yes 🞎 no |
| Constipation | 🞎 yes 🞎 no |
| Pruritus | 🞎 yes 🞎 no |
| Urticaria | 🞎 yes 🞎 no |
| Jaundice | 🞎 yes 🞎 no |
| Cough and chest pain | 🞎 yes 🞎 no |
| Sweating, dizziness | 🞎 yes 🞎 no |

*If yes, give antiemeticum

| **Physical examination** |  |
| --- | --- |
| Hepatomegaly |  |
| Abdominal tenderness |  |
| Splenomegaly |  |
|  |  |

| Anthelminthic treatment in the last 4 weeks |  | Other medication |
| --- | --- | --- |

**Parasitological stool survey** (Fasciola eggs/gram)

| Stool sample | Formal Ether sedimentation | Kato Katz 1 | Kato Katz 2 | Kato Katz 3 |
| --- | --- | --- | --- | --- |
| 1 |  |  |  |  |
| 2 |  |  |  |  |
| 3 |  |  |  |  |
|  |  |  |  |  |

| **Hematology and biochemistry** |  |  |
| --- | --- | --- |
| Erythrocyte sedimentation rate |  | |
| Red blood cell count |  | |
| White blood cell count |  | |
| Platelets |  | |
| Differential blood cell count |  | |
| Alkaline phosphatase |  | |
| Alanine amino transferase |  | |
| Gamma glutamyltransferase |  | |
| Bilirubin |  | |
| Urea |  | |
| Creatinine |  | |

**Drug administration**

| Administration of artemether | 🞎 |
| --- | --- |
| Patient name: | Patient No. |

Date/time_________________

Date/time_________________

Date/time_________________

**Appendix 2: Summary and grading of adverse events**
